# Expression and epitope prediction of MPT64 recombinant proteins from clinical isolates of *Mycobacterium tuberculosis* as immunoserodiagnostic candidates

**DOI:** 10.14202/vetworld.2022.2376-2383

**Published:** 2022-10-08

**Authors:** Fihiruddin Fihiruddin, Nurul Inayati, Raudatul Jannah, Lalu Unsunnidhal, Asmarani Kusumawati

**Affiliations:** 1 Department of Medical Laboratory Technology, Politeknik Kesehatan Mataram, Praburangkasari Street, Indonesia; 2 Center of Excellent, Politeknik Kesehatan Mataram, Praburangkasari Street, Indonesia; 3 Midwifery Study Program, STIKES Yarsi Mataram, West Nusa Tenggara, 83361, Indonesia; 4 Food Technology Study Program, Faculty of Food Technology and Agroindustry, University of Mataram, Mataram, 83125, Indonesia; 5 Biomedical Field, Nursing Study Program, STIKES Yarsi Mataram, West Nusa Tenggara 83361, Indonesia; 6 Department of Reproduction and Obstetrics, Faculty of Veterinary Medicine, Universitas Gadjah Mada, Yogyakarta 55281, Indonesia

**Keywords:** *Escherichia coli*, immune response, lymphocytes, tuberculosis

## Abstract

**Background and Aim:**

The success in the handling and prevention of tuberculosis (TB) cases is highly dependent on their rapid detection, monitoring, and treatment. The efficacy of the Bacille Calmette–Guerin (BCG) vaccine is inconclusive in eastern Indonesia. The *RV1980c* gene of *Mycobacterium tuberculosis* encodes an antigenic protein that is considered to be a virulence factor, as it can stimulate the immune response in patients with TB. This study aimed to study the expression and epitope indicator of MPT64 recombinant proteins from clinical isolates of *M. tuberculosis* as immunoserodiagnostic candidates for pET SUMO plasmids from clinical isolates as candidates for serodiagnostic tests and recombinant vaccines.

**Materials and Methods:**

The polymerase chain reaction (PCR) product of the *RV1980c* gene was inserted into the SUMO pET plasmid, which was then transformed into *Escherichia coli* BL21 (DE3) cells and expressed in Luria Bertani media induced by 1.0 M IPTG. Subsequently, sequencing was performed and the results were analyzed using the ClustalW and National Center for Biotechnology Information BLAST software. The T-cell epitope prognosis was then explained by GENETYX version 8.0., for the prediction of B-cell epitope, as assessed using an Immune Epitope Database analysis.

**Results:**

The PCR product of the *RV1980c* gene had a length of 619 bp. Moreover, SDS– polyacrylamide gel electrophoresis and Western blotting revealed that the protein encoded by the *Rv1980c* gene weighed 36 kDa. We gained nine specific T-cell epitopes according to Iad Pattern position and eight epitopes according to Rothbard/Taylor Pattern Position; furthermore, we detected five B-cell epitopes in the *RV1980c* gene.

**Conclusion:**

The MPT64 protein encoded by the *RV1980c* gene carries epitopes that are realized by lymphocytes and represent potential immunoserodiagnostic candidates in diagnostic immunology.

## Introduction

Tuberculosis (TB) is a very serious fitness concern and a risk factor for long-term respiratory impairment that needs treatment and attention [1], because it is responsible for millions of deaths per year worldwide [2], particularly in developing countries. The TB disease in humans is a directly contagious disease caused by *Mycobacterium* *tuberculosis*, which affects mainly the lungs, but can also attack other organs. In 2020, an estimated 10 million people fell ill with TB worldwide: 5.6 million men, 3.3 million women, and 1.1 million children [3].

Indonesia ranks second in the incidence of TB in the world, after India [4]. Because of its characteristics, such as slow growth and highly varying strains, *M. tuberculosis* is the main problem in TB treatment. Additional challenges include the presence of a resistant strain of TB (multidrug-resistant-TB [MDR-TB]), infection with the human immunodeficiency virus or the presence of autoimmune deficiency syndrome (HIV or AIDS), the presence of latent infection reaching 40–50% of infection cases, and decreased effectiveness of vaccines and diagnostic methods, which have generally been ineffective for the development of successful treatments [5–10].

Several strategies have been investigated for the control and elimination of TB. The search for more effective vaccine candidates, including vaccine subunits, is important, [7–12]. To date, Bacillus Calmette–Guerin (BCG) is the only TB vaccine that has been used in humans [13] worldwide since 1921 [14]. The BCG vaccine prevents infection in newborns [15, 16], but provides poor protection and highly variable efficacy against TB infection in adults [14]. The elimination of a large number of TB cases globally depends on the early detection, diagnosis, and handling of cases [17].

MPT64 is a protein that is secreted by *M. tuberculosis* in the early phase of infection that has the potential to be developed as a promising antigen tool for serodiagnostic testing [18] and recombinant DNA vaccine development [7–12, 15, 19]. MPT64 has a very dominant antigen that is recognized by T cells and plays a role as a virulence factor of *M. tuberculosis*. This protein transfers bacteria present in the phagosome into the cell cytoplasm at the time of initial infection [20]. MPT-64 strongly induces T cells and interferon-gamma (INF-γ) [21] because it carries epitopes that are remembered by T cells and B cells in patients. MPT-64 is encoded by the *RV1980c* gene and is found only among the virulence factors of *M. tuberculosis* [22, 23]. The production of recombinant proteins from the MPT64-encoding-gene could be used to develop a better diagnostic test or a combination of vaccines [7–12, 24].

The use of a plasmid as a carrier medium of genes to be expressed will determine the success of the formulation of these recombinant proteins. Each plasmid is equipped with different peptide tags. The pET SUMO vector is used as a recombinant plasmid to produce recombinant proteins efficiently. One of the advantages of using pET SUMO pET as a cloning vector is its ability to produce recombinant proteins without the presence of additional amino acids [7–12, 19, 25, 26]. Moreover, it does not require digestion using restricted enzymes to obtain the polymerase chain reaction (PCR) product [7–12, 26–28].

This study aimed to study the expression and epitope indicator of MPT64 recombinant proteins from clinical isolates of *M. tuberculosis* as immunoserodiagnostic candidates for pET SUMO plasmids from clinical isolates as candidates for serodiagnostic tests and recombinant vaccines.

## Materials and Methods

### Ethical approval

Ethical clearance to obtain samples of *M. tuberculosis* from patients at the Balai Besar Laboratorium Kesehatan (BBLK) was obtained from the Ethical Clearance Commission of the Faculty of Veterinary Medicine, Gadjah Mada University (Approval Number: 0021/EC-FKH/Eks/2018).

### Study period and location

The study was conducted from December 2020 to November 2021. The study was conducted at the Balai Besar Laboratorium Kesehatan (BBLK) Surabaya, and the patients were followed at Dr. Sutomo Hospital in Surabaya and East Java area.

### DNA extraction and concentration assessment

Isolates of *M. tuberculosis* were obtained from patients that were recognized as having lung disease caused by MDR-TB. The bacterial control used was the *M. tuberculosis* strain H37Rv. The recognition of *M. tuberculosis* was performed using a BACTEC MGIT 960 System (BD) and detection of the bacterial antigen of M. tuberculosis was achieved using the TB Ag MPT 64 Rapid test (SD Bioline, Korea) at the microbiology laboratory of BBLK Surabaya. Drug-resistance tests against TB were accomplished on Lowenstein medium and validated using a GeneXpert® System test (Cepheid, USA). The isolates of *M. tuberculosi*s and MDR-TB were subcultured (refreshed) in the microbiology laboratory of BBLK Surabaya, to obtain isolates in about 3 weeks of the log phase of growth, whereas the molecular test was conducted on isolates with a 3-week average growth. The process of extracting *M. tuberculosis* from the medium was carried out using the DNeasy Blood and Tissue Kit from Qiagen (Germany). The compacting of the extracted bacterial DNA was measured using a NanoDropTM2000/2000c Spectrophotometer (Chongqing Drawell Instrument Co. Ltd., China).

### Layout of the PCR

The region to be amplified was resolved based on the sequence of *M. tuberculosis* H37Rv (ATCC 27294). The primers used were as follows: forward primer: 5′-*GCG CCC AAG ACC TAC TGC GAG GAG* -3’, reverse primer: 5’- *CTA GGC CAG CAT CGA GTC GAT CGC* -3’. Amplification of the MPT64-encoding gene was performed using a PCR Amplitron machine (Thermolyne, Thermo Fisher Scientific, USA) in a final reaction volume of 25 μL, including 12.5 μL of the PCR mix, 2 μL of the forward primer, 2 μL of the reverse primer, 6.5 μL of nuclease-free water, and 2 μL of the template. Optimization was performed using the following conditions: pre-denaturation for 5 min at 95°C; followed by 35 cycles of denaturation for 30 s at 95°C, annealing for 30 s at 56°C, and extension for 1 min at 72°C; and a final extension for 10 min at 72°C, followed by cooling for 10 min. The PCR products were processed by electrophoresis and their inspection was conducted under ultraviolet light, which revealed specific bands for *RV1980c* at 619 bp.

### Cloning and transformation

The process of ligation was performed by mixing plasmid DNA with fresh PCR product inside a tube containing the following elements: 2 μL of fresh PCR product, 1 μL of 10× ligation buffer, 2 μL of pET SUMO Vector, 4 μL of sterile water, and 1 μL ofT4 DNA ligase. The ligation products were stored at –20°C, ready for transformation (Champion™ pET SUMO, Thermo Fisher Scientific, USA). The transformation was carried out by taking the blue tube (One Short^®^Mach1TM-T1R, Thermo Fisher Scientific, USA) and placing it in an ice container, allowing it to melt, and adding 10 μL of plasmid from the ligation step. This was then mixed until homogeneity was reached without using a vortex, followed by incubation and cell heat shock at 42°C and immediate storage in a tube on ice. Next, 250 μL of SOC medium was added and the tube was closed tightly and incubated at 37°C for 1 h in a shaker at 200 rpm. The products of the transformation step were then sprayed onto Luria Bertani (LB) agar media (50, 100, and 150 μL each in LB plate medium on a Petri dish with a cover slip), then incubated at 37°C for 24–48 h. The expansion of colonies in the LB medium was observed and plasmid DNA isolation was performed using a PureLinkTM HQ Mini Plasmid Purification Kit (Thermo Fisher Scientific, USA). Transformation for the expression of proteins was carried out by taking a chocolate cap tube (competent *Escherichia coli* BL21 [DE3] One Short® cells, Thermo Fisher) and adding 5–10 ng of plasmid DNA. This was mixed using a pipette tip and incubated on ice for 30 min, and the cells were heat shocked for 30 s at 42°C, then suddenly stored on ice in a tube. Next, 250 μL of SOC medium was added and the tube was closed tightly and incubated at 37°C for 1 h in a shaker at 200 rpm. Finally, the transformation reaction was ready for transfer to an LB agar plate.

### Recombinant protein test by Sodium dodecyl-sulfate–polyacrylamide gel electrophoresis (SDS–PAGE)

An MPT64 pure recombinant protein was obtained by nickel column chromatography (Ni-NTA Purification System, Thermo Fisher Scientific Inc., Massachusetts, USA). The preparation of a 15% gel gradient and 3% gel stacking was performed for protein electrophoresis. The polymerization of the gel gradient occurred over the next 4–5 h. The surface of the gradient gel was cleaned with Aquadest produced by Biochemistry Laboratory, Research Center for Biotechnology, Gadjah Mada University, Yogyakarta, Indonesia to remove the remains of butanol. The specimens were collected and a dye was added to the tube at a ratio of 10 μL of dye to 40 μL of the specimen, followed by homogenization and heating to the boiling point of water at 80°C for 5 min. The sample was allowed to cool and was then placed in the electrophoresis well. Electrophoresis was achieved at 120 V for 2 h. After completion of the electrophoresis step, the gel was collected and stained. The gel was cleaned using Aquabidest produced by Biochemistry Laboratory, Research Center for Biotechnology, Gadjah Mada University, Yogyakarta, Indonesia, followed by a destaining solvent produced by Biochemistry Laboratory, Research Center for Biotechnology, Gadjah Mada University, Yogyakarta, Indonesia (30% methanol and 10% acetic acid solution) for 30 min, until the band was clearly visible, and was then cleaned with water, to remove any remaining color.

### Western blotting of recombinant MPT64 proteins

The protein bands obtained through SDS–PAGE were electrotransferred onto a polyvinylidene difluoride (PVDF) membrane at 500 mA for 2 h. Next, the membrane was blocked using 1% serum bovine albumin, followed by incubation with a monoclonal primary antibody and the corresponding secondary antibody. The reaction was stopped by the addition of chemicals, namely, nitro blue tetrazolium/5-bromo-4-chloro-3-indolyl-phosphate (NBT/BCIP) substrates.

## Results

**Figure-1:**
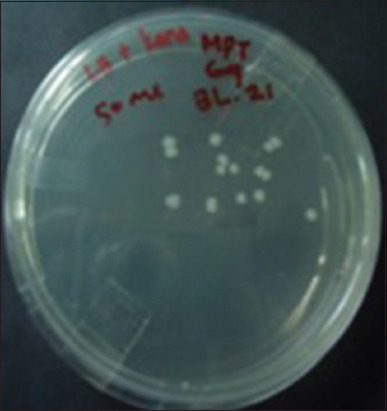
The growing of competent cells of *Escherichia coli* BL21 (DE3) One Short^®^ cells, (Thermo Fisher Scientific Inc., Massachusetts, USA) on LB agar. The colonies of *E. coli* competent cells with a pET SUMO plasmid inserted to RV1980c *gene* of *Mycobacterium tuberculosis*.

**Figure-2:**
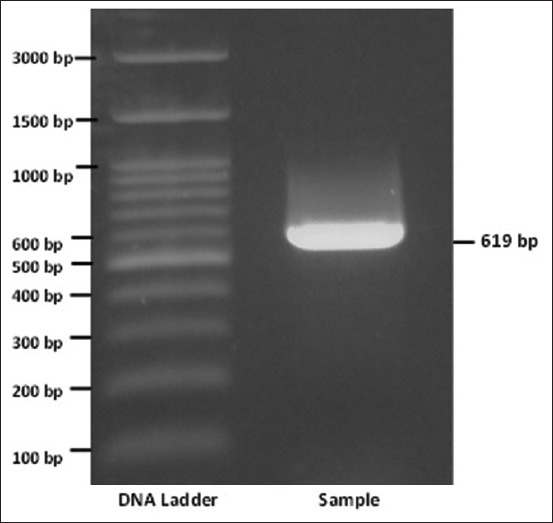
Results of polymerase chain reaction from bacterial colonies grown on Luria Bertani agar medium. Line 1: DNA ladder, line 2: RV1980c gene (619 bp) of *Mycobacterium tuberculosis*.

The recombinant plasmid containing the inserted gene encoding the MPT-64 protein was substantially transformed into competent *E. coli* BL21 cells (Figure-1). The amplification of the gene encoding the MPT-64 protein produced a single fragment with a length of 619 bp (Figure-2). The result of the amplification of the gene encoding the MPT64 protein through PCR of the fresh produce was used for DNA insertion in the ligation process. The ligation process used the pET SUMO vector as a cloning vector. This vector was designed to facilitate the cloning of PCR products directly. The pET SUMO vector has a nucleotide size of 5643 bp. The amplified gene that encoded the MPT64 protein from the competent *E. coli* BL21 (DE3) cells (Figure-2) was successfully inserted in the pET SUMO plasmid. The amplification of the gene insertion region using the primers of the plasmid (SUMO protease forward and T7 reverse primers) revealed the size of the gene that was inserted according to the length of the insertion. The compatibility of the insertion gene size from the PCR examination results of bacterial colonies grown on LB medium was confirmed by sequencing.

The results of the sequencing of the *Rv1980c* gene of *M. tuberculosis* after processing using the ClustalW program showed a similarity level of 100% with the original sequence of the *M. tuberculosis* *H37Rv* gene. The *Rv1980c* gene sequences from this alignment did not exhibit similarity with non-TB mycobacteria (Table-1).

**Table-1:** Homology of specimen isolate with bacteria in NCBI database.

No.	Bacterial species	Query (%)	Coverage expected value	Identity (%)
1.	*M. tuberculosis R2092*	100	0.0	100
2.	*M. tuberculosis H37Rv*	100	0.0	100
3.	*M. tuberculosis CG24*	100	0.0	100
4.	*M. tuberculosis CG21*	100	0.0	100
5.	*M. tuberculosis CG20*	100	0.0	100
6.	*M. tuberculosis strain 1-0006P6C4*	100	0.0	100
7.	*M. tuberculosis strain 1-0007P6C4*	100	0.0	100
8.	*M. tuberculosis strain 1-009P6C4*	100	0.0	100
9.	*M. tuberculosis strain 1-0013P6C4*	100	0.0	100
10.	*M. tuberculosis strain 1-0017P6C4*	100	0.0	100
11.	*M. tuberculosis varian bovis BCG* and *strain BCG SL222*	100	0.0	100

NCBI=National Center for Biotechnology Information, BCG=Bacille Calmette–Guerin, *M. tuberculosis*=*Mycobacterium tuberculosis*

The prediction of the MPT64 recombinant protein epitope against T cells was carried out using the GENETYX software, version 8.0. Prediction of T-cell epitopes of MPT64 proteins by the Iad Pattern position method and the Rothbard/Taylor Pattern position method yielded 9 and 8 epitope positions, respectively (Table-2). Prediction of the MPT64 recombinant protein epitope against B cells was performed using an Immune Epitope Database (IEDB) analysis. The epitope prediction results for B cells showed that the MPT64 protein had 5 epitope positions (Table-3).

**Table-2:** Epitope prediction of MPT64 recombinat proteins of *M. tuberculosis* againts T cells using GENETYX.ver. 8.0 program.

Gene	T cell epitope			

	Iad Pattern Position		Rothbard or Taylor Pattern Position	
	
	Amino acid with position	Sequence	Amino acid position	Sequence
*Rv1980c*	9–14	LKGTDT	26–29	DPAY
	57–62	LSAATS	55–58	KFLS
	59–64	AATSST	67–70	EAPY
	60–65	ATSSTP	109–112	KAFD
	72–77	LNITSA	119–122	KPIT
	77–82	ATYQSA	162–165	DPVN
	85–90	PRGTQA	184–188	ELLPE
	105–110	TTTYKA	188–191	EAAG
	195–200	VLVPRS		

*M. tuberculosis*=*Mycobacterium tuberculosis*

**Table-3:** Epitope prediction of MPT64 recombinant proteins of *M. tuberculosis* againts B cells using *IEDB Analysis* program.

No.	Start	End	Peptide	Length
1	7	18	EELKGTDTGQAC	12
2	38	47	YYPDQKSLEN	10
3	60	70	ATSSTPREAPY	11
4	116	132	AYRKPITYDTLWQADTD	17
5	147	165	KQTGQQVSIAPNAGLDPVN	19

*M. tuberculosis*=*Mycobacterium tuberculosis*, IEDB=Immune Epitope Database

**Figure-3:**
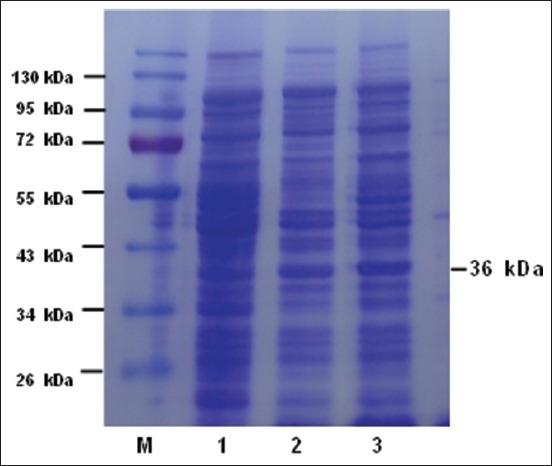
Sodium dodecyl sulfate–polyacrylamide gel electrophoresis of refined MPT64 proteins pET SUMO clone in *Escherichia coli* BL21 (DE3). Line 1: Protein ladder and line 2, 3, and 4: Crude MPT64-proteins (36 kDa).

**Figure-4:**
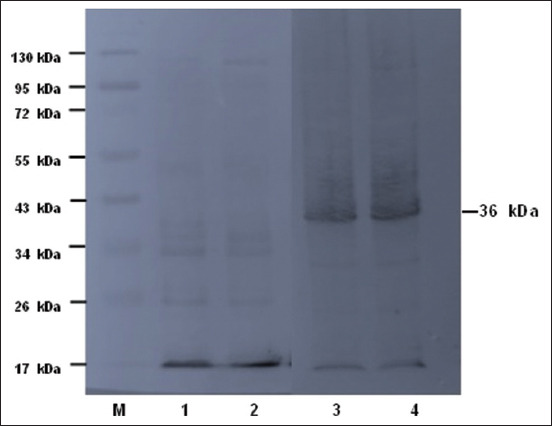
Western blotting of MPT64 proteins using 6× anti-histidine Tag monoclonal antibody. Line 1: Protein ladder, Line 2 and 3: Negative control, Line 4 and 5: A purified MPT-64 proteins (36 kDa).

The results of the electrophoresis of the recombinant protein product through SDS–PAGE followed by Western blotting (WB) using an anti-histidine-tag monoclonal antibody (6×) are presented in Figures-3 and 4. The recombinant proteins obtained from cultures and IPTG induction optimization were visualized in SDS–PAGE using a gel gradient of 12% for the resolving gel and 5% for the stacking gel (Figure-3). The WB of recombinant proteins was visualized using PVDF membranes (Figure-4).

## Discussion

We studied the expression of the recombinant MPT64 protein epitopes from clinical isolates of *M. tuberculosis* as immunoserodiagnostic candidates using the pET SUMO plasmid from clinical isolates as serodiagnostic test candidates and recombinant vaccines. This was achieved using recombinant techniques, which allow the expression of genes of interest *in vivo* using competent *E. coli* cells. These bacteria are widely used for the overexpression of recombinant proteins. *E. coli* has a high transformation efficiency, grows at a fast rate, affords relatively simple plasmid preparation, and produces DNA products at a high level [7–12, 19, 29, 30]. In some instances, the expression of proteins in medium through the extracellular expression system yields a small amount of product. In the previous research, the expression of the MPT64 protein was carried out on *E. coli* BL21(DE3) cells extracellularly [31]. The protein expression results obtained remained relatively modest and recombinant MPT64 protein was detected in the cytosol (intracellularly) and in the periplasmic area [31].

An investigation of MPT64 was conducted and used to determine the ideal sensitivity and specificity for detecting *M. tuberculosis* either in single or recombinant antigens [23]. In this study, the gene encoding MPT64 in *M. tuberculosis* was successfully propagated using specific primers and designed to target the *M. tuberculosis* *H37Rv* genome. The clinical isolates of patients with TB yielded bands according to the target with a nucleotide size of 619 bp.

The sequencing data confirmed that each gene inserted into the pET SUMO plasmid showed a size that was compatible with the inserted fragment. Cloning was carried out by inserting the PCR products with amplified genes encoding *M. tuberculosis* MPT64 into the pET SUMO vector according to the Champion™ pET protocol of SUMO Protein Expression Process (Invitrogen) kits. A plasmid gene in the ligation system was not necessary because the pET SUMO vector has a T overhang sequence design. The Taq polymerase used to obtain the PCR product has a non-template-dependent activity and added a single deoxyadenosine (A) at the 3′ end. The pET SUMO vector includes a single deoxythymidine (T) residue for binding to the single deoxyadenosine (A) present in the PCR product. The gene encoding the *M. tuberculosis* MPT64 protein was successfully inserted into the SUMO pET vector and transformed into competent *E. coli* BL21 cells. The growth of transformants on LB plate medium with the addition of kanamycin at 50 μg/mL yielded large amounts of bacterial colonies. *Escherichia coli* BL21 colony growth on selection medium with kanamycin was promoted by *E. coli* BL21 bacteria carried by the pET SUMO vector. This vector has a kanamycin-resistant marker gene.

The screening tests of several colonies of competent *E. coli* BL21 (DE3) cells grown on LB medium showed that amplification with specific primers for the MPT64-encoding *RV1980c* gene yielded specific bands sized at 619 bp. The positive DNA plasmid carrying the MPT64-encoding gene was confirmed using SUMO forward and T7 reverse primers, to detect the inserted gene in the right frame [28]. Several amplified colonies showed that the gene encoding the MPT64 protein, which was inserted into the pET SUMO vector, was transformed well into competent cells. This study also sequenced the inserted genes.

The sequence results were analyzed and an alignment was performed using the ClustalW program, which showed that the inserts of the gene encoding MPT64 were in the appropriate frame and there was no change in the position or exchanges between the deoxyadenosine start codon (A) and the deoxyadenosine stop codon (A) that was bound within the deoxythymidine (T) on the TA cloning site. Positive bacterial colonies carrying the inserted gene in the appropriate frame were used for further analysis of recombinant proteins.

The bands of the recombinant MPT64 fusion SUMO protein obtained from competent *E. coli* BL21 (DE3) bacterial cell pellet induced by 1 mM IPTG were visualized on SDS–PAGE and showed that these bacteria were able to express MPT64 recombinant proteins. The visualization of recombinant MPT64 proteins using SDS–PAGE revealed bands at a molecular weight of 36 kDa., whereas the molecular weight of the non-recombinant MPT64 protein was 24 kDa. The increased molecular weight observed was attributed to the addition of tag proteins (12 kDa) from the pET SUMO plasmid.

Furthermore, to confirm the expression of the proteins, WB using an anti-his-tag antibody (mouse monoclonal) was performed. In turn, the anti-his-tag antibody was detected with anti-mouse IgG antibodies. The bands on the PVDF membrane were visualized after adding a 1-step NBT/BCIP substrate solution at 36-kDa positions. Previously, the results of MPT64 protein expression [20] showed that this protein has 228 amino acids and a molecular weight of 24.8 kDa. This protein is an immunodominant antigen that is very important for superoxide dismutase activity and very strong cellular immune responses. At the *RD2* locus, the *Rv1980c* gene is a part of the gene complex that encodes a continuous cell wall protein of *M. tuberculosis* [23]. The expression of these genes produces proteins that play an important role in the virulence of *M. tuberculosis* [21].

The MPT64 protein is encoded by the *RV1980c* gene and is secreted into the endoplasmic reticulum. The secreted MPT64 protein can induce the lysis of the phagosomic membrane from macrophages. MPT64 is among the proteins that are secreted early in the onset of TB infection and plays a role in adhesion, invasion, and cell lysis in macrophages that act as virulence factors [32], because they can modulate the immune response by producing INF-γ and stimulating T cells, thus causing delayed-type hypersensitivity reactions [33], overexpression of pro-inflammatory cytokines, and the production of reactive oxygen species (ROS) [21].

Tests using MPT64 recombinant proteins suggest that the combined use of MPT64 may provide a screening strategy for the development of TB serodiagnostic tests and anti-TB vaccines [24]. The prediction of T-cell epitopes of the MPT64 protein via the Iad Pattern position method and the Rothbard/Taylor Pattern position method revealed 9 and 8 epitope positions, respectively. In turn, MPT64 protein epitope prediction based on an IEDB analysis identified 5 epitope positions.

The results of the previous studies showed that there were differences in the epitope prediction for the MPT64 protein [24, 34]; 23 positions of epitopes against T cells were identified on the MPT64 antigen based on IEDB analysis, half (52.17%) of which had amino acid changes. The deletion of the 63-bp sequence affected 5 of the 23 T-cell epitopes in the antigen (61834, 53370, 33402, 28594, and 75172). These conditions suggest that the expression of this gene may trigger varying immune responses [16]. There are three point mutations in the *Rv1980c* gene, namely, at nucleotide positions 3, 26, and 37 [35]. The difference in the number and position of T-cell epitopes that have been found in this study is attributed to the primers used here. Moreover, this difference may also have been caused by the use of a different program to predict T-cell epitopes.

## Conclusion

The gene encoding the MPT64 protein of *M. tuberculosis* from clinical isolates of patients was successfully inserted after amplification into the pET SUMO vector and transformed into competent *E. coli* BL21 (DE3) cells, yielding a product with a size of 619 bp and a protein with a molecular weight of 36 kDa. T-cell epitope prediction for the MPT64 protein using the GENETYX 8.0 software based on Iad Pattern position and Rothbard/Taylor Pattern Position identified 9 and 8 epitope positions, respectively. Prediction of B-cell epitopes using an IEDB analysis found 5 epitopes. MPT64 may provide a screening strategy for the development of candidates for serodiagnostic tests and recombinant vaccines against *M. tuberculosis*.

## Authors’ Contributions

FF: Led the research, supervised the overall research work, drafted the manuscript, and revised intellectual content. LU, NI, RJ, and AK: Participated in sampling, made available relevant literature, collection of data, executed the experiment, interpretation of the results, and drafted the manuscript. All authors have read and approved the final manuscript.
